# Chromosome-level genome assembly of the yellow-cheek carp *Elopichthys bambusa*

**DOI:** 10.1038/s41597-024-03262-8

**Published:** 2024-04-24

**Authors:** Shunyao Li, Xuemei Xiong, Siyu Qiu, Zhigang Shen, Yan He, Zexia Gao, Shiming Wan

**Affiliations:** 1 College of Fisheries, Key Lab of Freshwater Animal Breeding, Ministry of Agriculture/Key Lab of Agricultural Animal Genetics, Breeding and Reproduction of Ministry of Education/Engineering Research Center of Green development for Conventional Aquatic Biological Industry in the Yangtze River Economic Belt, Ministry of Education/Engineering Technology Research Center for Fish Breeding and Culture in Hubei Province, Huazhong Agricultural University, Wuhan, 430070 China; 2 Hubei Hongshan Laboratory, Wuhan, 430070 China

**Keywords:** Genome, Ichthyology, Animal breeding

## Abstract

Yellow-cheek carp (*Elopichthys bambusa*) is a typical large and ferocious carnivorous fish endemic to East Asia, with high growth rate, nutritional value and economic value. In this study, a chromosome-level genome of yellow-cheek carp was generated by combining PacBio reads, Illumina reads and Hi-C data. The genome size is 827.63 Mb with a scaffold N50 size of 33.65 Mb, and 99.51% (823.61 Mb) of the assembled sequences were anchored to 24 pseudo-chromosomes. The genome is predicted to contain 24,153 protein-coding genes, with 95.54% having functional annotations. Repeat elements account for approximately 55.17% of the genomic landscape. The completeness of yellow-cheek carp genome assembly is highlighted by a BUSCO score of 98.4%. This genome will help us understand the genetic diversity of yellow-cheek carp and facilitate its conservation planning.

## Background & Summary

Yellow-cheek carp (*Elopichthys bambusa*), also known as “water tiger”, is a species in the order *Elopichthys*, subfamily Leuciscinae and family Cyprinidae. Yellow-cheek carp is a typical large and ferocious carnivorous fish endemic to East Asia. In China, it is mainly distributed in river systems such as the Yangtze River, Pearl River and Yellow River^1^. Yellow-cheek carp lives in the upper layer of rivers and lakes, it has a strong swimming ability and chases other fish for food. Yellow-cheek carp can prey on diseased and weak fish to control their population size, which is of great significance for maintaining the ecological balance of the water environment^2^. Yellow-cheek carp is also an important characteristic economic fish with firm meat, delicious taste, and rich in high-quality protein, unsaturated fatty acids, minerals and other nutrients^3–5^. However, anthropic factors such as overfishing, hydrological modification and water pollution have led to the dwindling natural resources of yellow-cheek carp^6,7^, which has been listed in the “Key Protected Endangered and Threatened Aquatic Species” and the IUCN Red List of Threatened Species (Version 2020.3)^8^.

The typical carnivorous yellow-cheek carp is particularly special among East Asian carp species that are mainly omnivorous and herbivorous. For example, yellow-cheeked carp and grass carp both belong to the subfamily Leuciscinae and had the closest relationship. Interestingly, they have evolved completely opposite feeding habits^9^, which provides excellent material for studying the evolution and genetic regulation mechanisms of fish feeding habits. However, the lack of genomic information limits the study on the carnivorous formation mechanism of yellow-cheek carp. At the same time, higher breeding profits have also promoted the continuous development of the artificial breeding industry of yellow-cheek carp. Using live fish or frozen fish as the main bait not only results in higher breeding costs for yellow-cheeked carp, but also easily causes pollution of the aquaculture water, which greatly restricts the expansion of the farming scale^10^. Therefore, research on the dietary transformation of typical carnivorous fishes such as yellow-cheek carp has gradually become a hot topic, and there is an urgent need for genetic breeding of yellow-cheek carp based on whole-genome information.

**Fig. 1:**
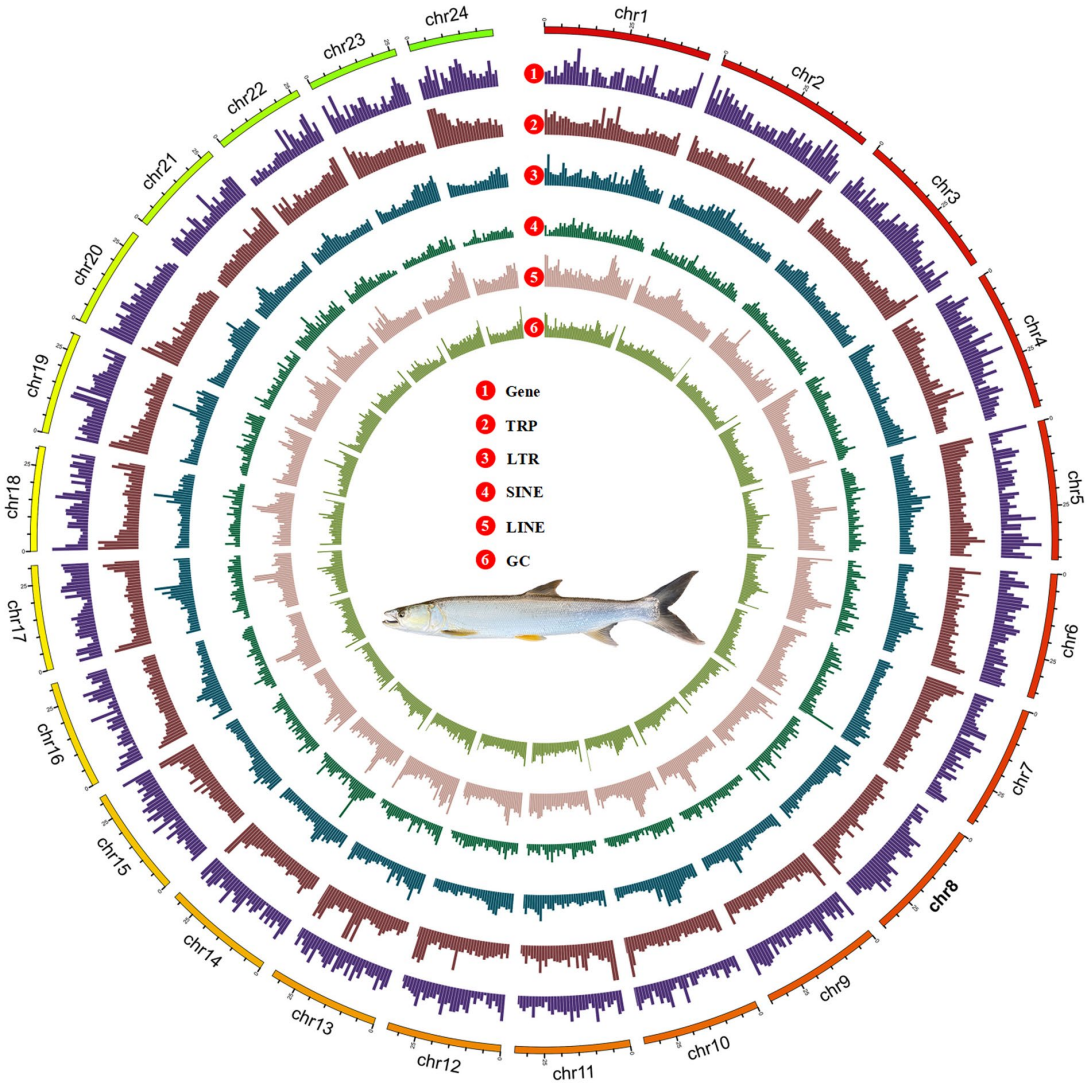
Characterization of assembled yellow-cheek carp genome. Circos plot of the yellow-cheek carp genome, with visualization of gene density (1), TRP (2), LTR (3), SINE (4), LINE (5) and GC content (6) in order from outside to inside.

In this research, we have combined PacBio long-read sequencing, Illumina short-read sequencing and Hi-C technology to generate a high-quality chromosome-level genome of the yellow-cheek carp (Fig. 1). Accordingly, we expect rapid progress in the genetics research of yellow-cheeked carp, and functional genes related to key economic traits of yellow-cheeked carp will continue to be discovered. The elucidation of the genome structures and functions will promote more in-depth research to better understand the genetic basis for the formation of important traits such as the carnivorous in yellow-cheeked carp, thereby making contributions to its resource protection, genetic selection and artificial breeding.

## Methods

### Sample collection and sequencing

An adult male yellow-cheek carp was collected from the Yangtze River in Wuhan, Hubei, China. High-quality genomic DNA was extracted from muscle by the CTAB method for Illumina sequencing, PacBio SMRT sequencing^11^ and Hi-C. The quality of the extracted DNA was assessed using agarose gel electrophoresis and NanoDrop Spectrophotometer (Thermo Fisher Scientific, USA), and quantified by a Qubit Fluorometer (Invitrogen, USA).

For Illumina sequencing, the genomic DNA was randomly sheared to 300~500 bp fragments, and a paired-end genomic library was prepared following the manufacturer’s protocol. Then, the library was sequenced on an Illumina NovaSeq platform using a paired-end 150 bp layout to enable genome survey and base-level correction. For PacBio long-read sequencing, SMRTbell libraries were constructed using the genomic DNA and sequenced on the PacBio Sequel II sequencing platform. After, approximately 58.98 Gb of Illumina short-read data (coverage of 71.31×) and 27.35 Gb of PacBio continuous long reads (CLR) data (coverage of 32.65×) was obtained.

To generate a chromosomal-level assembly of the yellow-cheek carp genome, a Hi-C library was generated using the DNA extracted from the same yellow-cheek carp. After cell crosslinking, cell lysis, chromatin digestion, biotin labelling, proximal chromatin DNA ligation and DNA purification, the resulting Hi-C library was subjected to paired-end sequencing with 150 bp read lengths on an Illumina NovaSeq platform. Finally, the size of Hi-C data obtained was 151.98 Gb, covering 183.78× of the genome.

To aid genome annotation, the total RNA from muscle, spleen, gonad and skin was extracted and tested for purity and integrity using a NanoDrop Spectrophotometer (Thermo Fisher Scientific, USA) and Agilent 2100 bioanalyzer (Agilent Technologies, USA). The RNA library was constructed using the NEBNext® UltraTM RNA Library Prep Kit (Illumina, USA) following the manufacturer’s protocol and sequenced on an Illumina NovaSeq. 6000 platform. Finally, 23.74 Gb of data was obtained (Table 1).

**Table 1:** Statistics of the sequencing data used for genome assembly.

Libraries	Insert sizes	Clean data (bp)	Sequencing coverage (×)
Illumina	300 bp	58,975,349,100	71.31×
PacBio	10–15 kb	27,351,494,268	32.65×
Hi-C	300 bp	151,983,658,870	183.78×
RNA	300 bp	23,735,378,400	27.81×

### Genome assembly

First, SOAPnuke (v2.1.0)^12^ was used to perform quality control of Illumina data, and the clean data were utilized for genome size estimation. K-mer analysis^13^ was conducted using GCE (v1.0.2). As a result, the genome size was estimated to be 786.16 Mb, with a heterozygosity ratio of 0.47% and repeat sequence ratio of 47.03% (Table 2). A total of 27.35 Gb PacBio long-read data were used for de novo genome assembly using MECAT2 (v2.0.0)^14^ and NextDenovo (v2.4.0). The polishing was then carried out by the software gcpp (v2.0.2) and pilon (v1.22)^15^. Based on these sequencing data, the resulting assembly consists of 170 contigs and has a total length of 827.63 Mb (Table 3).

**Table 2:** K-mer frequency and genome size evaluation of yellow-cheek carp genome.

K-mer number	K-mer Depth	Genome Size (Mb)	Heterozygous Ratio (%)	Repeat (%)
52,684,645,196	64	786.16	0.47	47.03

**Table 3:** Statistics for Hi-C assisted assembly.

	Total	Contig Num	Contig N50	Scaffold Num	Scaffold N50	Proportion	GC-percent
Hi-C assisted pre-assembly	827,626,473	170	9,879,208	—	—	—	—
Hi-C-assisted assembly	823,606,315	165	9,879,208	24	33,649,237	99.51%	37.45

### Hi-C scaffolding

**Fig. 2:**
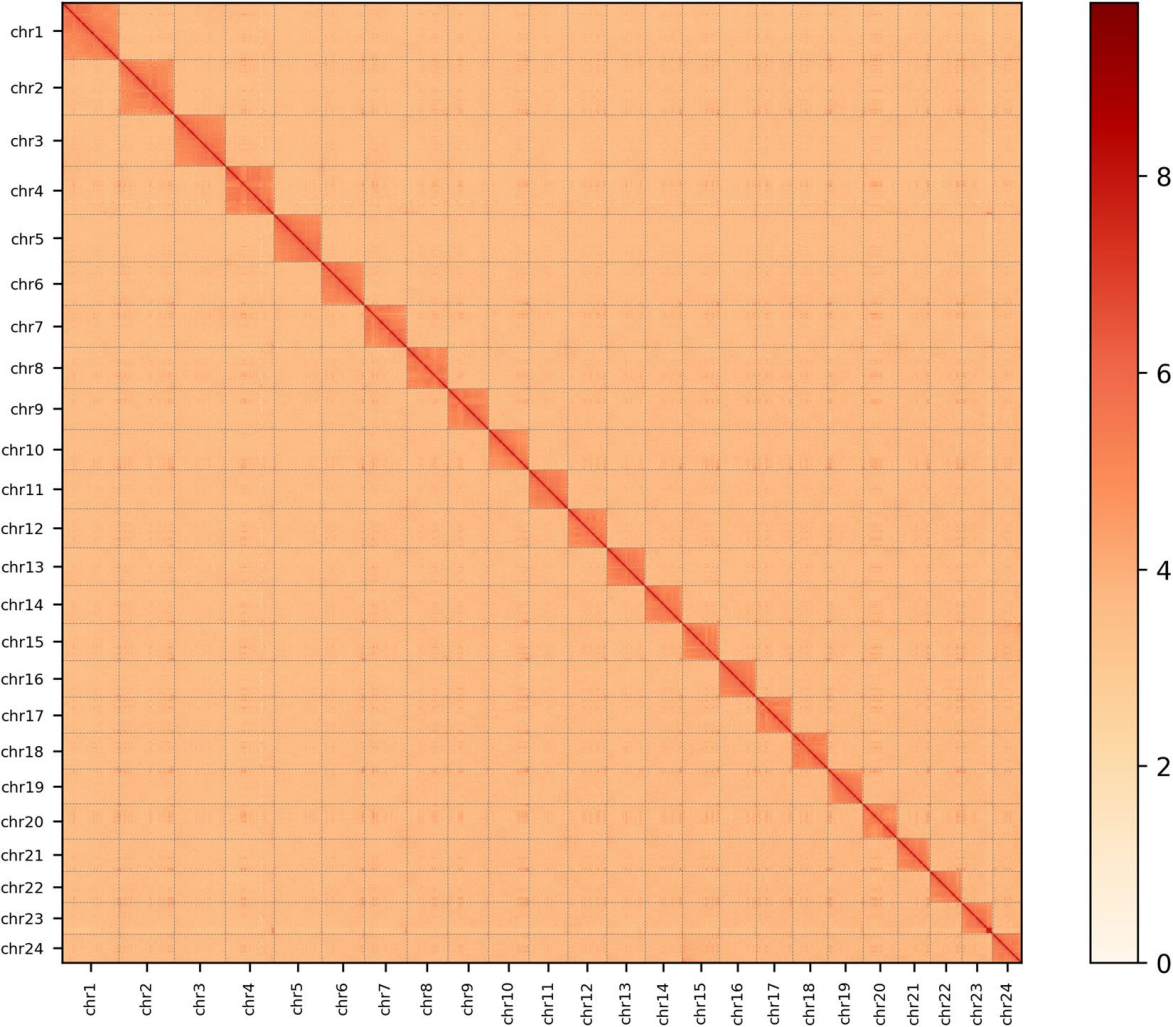
Genome-wide Hi-C interaction mapping of chromosome sections.

The Hi-C technology was used for chromosome-level genome assembly. The Trimmomatic^16^ with parameters (LEADING:3 TRAILING:3 SLIDINGWINDOW:4:15 MINLEN:50) was used to remove adapters and low-quality fragments of the raw Hi-C reads data. The processed reads were then aligned to the assembly using the Juicer (v1.6)^17^ with default settings. Contigs were scaffolded using 3D-DNA pipeline^18^ with all valid Hi-C reads. We use the Juicebox (v2.13.07)^17^ to adjust the chromosome-scale scaffolds manually(Fig. 2, Table 4). And there are 141 gaps among the 24 chromosomes.

**Table 4:** Chromosome and reference genome corresponding chromosome statistical results.

Chromosome ID	Number of Contigs	Length (bp)	Gaps
chr1	10	48,801,470	9
chr2	3	47,476,723	2
chr3	15	43,850,734	14
chr4	14	41,595,563	13
chr5	7	40,868,316	6
chr6	6	36,732,165	5
chr7	8	36,442,319	7
chr8	4	35,157,168	3
chr9	8	35,141,945	7
chr10	4	34,436,776	3
chr11	4	33,649,237	3
chr12	6	33,538,482	5
chr13	7	32,527,850	6
chr14	9	32,137,104	8
chr15	5	31,940,173	4
chr16	3	31,691,200	2
chr17	5	30,801,312	4
chr18	8	30,664,716	7
chr19	3	30,038,157	2
chr20	9	29,852,686	8
chr21	7	27,984,395	6
chr22	5	26,913,480	4
chr23	10	26,690,801	9
chr24	5	24,744,043	4
TOTAL	165	823,676,815	141

### Repeat annotation

We used de novo prediction and homology comparison to annotate the genomic repetitive sequences. RepeatModeler^19^ were used to detected and classified the repetitive sequences in the genome assembly using tools including RECON(v1.08)^20^, RepeatScout(v1.0.5)^21^, LTR-FINDER(v1.0.5)^22^ and TRF (v4.0.935)^23^. For homology comparison, RepeatMasker (open-4.0.9) and RepeatProteinMask (open-4.0.9) were used to identify the known TEs of the yellow-cheek carp genome in the Repbase TE library^24,25^ and TE protein database, respectively. The results showed that the genome repetitive sequence size was 456.66 Mb, accounting for 55.17% of the assembled genome. Among the repeat elements, short interspersed nuclear elements (SINEs) accounted for 0.24% of genome size and long interspersed nuclear elements (LINEs) accounted for 7.67%. Long terminal repeats (LTRs) and DNA elements accounted for 12.31% and 34.87%, respectively (Table 5).

**Table 5:** Repetitive elements and their proportions in yellow-cheek carp genome.

Type	Repbase TEs		Protein TEs		Denovo TEs		Combined TEs	
	Length (bp)	Percentage (%)	Length (bp)	Percentage (%)	Length (bp)	Percentage (%)	Length (bp)	Percentage (%)
**DNA**	135,569,082	16.38	21,468,489	2.59	208,673,761	25.21	288,628,347	34.87
**LINE**	17,380,180	2.1	17,851,894	2.16	52,066,672	6.29	63,480,091	7.67
**SINE**	1,034,564	0.12	0	0	1,364,468	0.16	2,016,734	0.24
**LTR**	24,846,205	3	19,281,719	2.33	91,771,796	11.09	101,898,770	12.31
**Unknow**	18,87,900	0.23	6,603	0	44,616,285	5.39	46,455,288	5.61
**Total**	173,959,113	21.02	58,476,207	7.06	343,673,320	41.52	429,931,954	51.94

### Protein-coding gene prediction and annotation

In this research, the *ab initio* gene prediction, homology-based gene prediction and transcript prediction were used to predicted protein-coding genes of the yellow-cheek carp genome. Prior to gene prediction, the assembled yellow-cheek carp genome was hard and soft masked using RepeatMasker. The *ab initio* gene prediction was performed using Augustus (v3.3.1)^26,27^ and Genescan (v1.0)^28^. Models used for each gene predictor were trained from a set of high-quality proteins generated from the RNA-Seq data. For the homology-based prediction, Glimmer HMM(v3.0.4)^29^ was used to align the protein sequences to our genome assembly and predict coding genes with the default parameters. The reference protein sequences of five fish species, including *Ctenopharyngodon idella*, *Sinocyclocheilus grahami*, *Megalobrama amblycephala*, *Danio rerio* and *Cyprinus carpio*, were sourced from the NCBI database. For the transcript prediction, clean RNA-Seq reads were assembled into the yellow-cheek carp genome using Stringtie (v2.1.1)^30^. Then the gene structure was formed using PASA (v2.4.1)^31^. To consolidate the results from these three methods, MAKER (v3.00)^32^ was employed to enable the merging and integration of gene predictions.

For functional annotation of predicted gene, BLASTP (v2.6.0)^33,34^ was used to align the anticipated genes to the Kyoto Encyclopedia of Genes and Genomes (KEGG)^35^, Gene Ontology (GO)^36^, NCBI-NR (non-redundant protein database), Swiss-Prot^37^, TrEMBL^38^ and InterPro^39^ database. In total, we successfully predicted 24,153 protein-coding genes within the genome. These predicted genes displayed an average coding sequence length of 1638.21 bp, an average gene length of 18969.98 bp, and an average exon number of 9.87 (Table 6). Further, 22,965 genes, which accounts for 95.54% of the total number of predicted genes, were successfully assigned with at least one functional annotation (Table 7).

**Table 6:** Basic statistical results of gene prediction.

Gene set	Number	Average gene length (bp)	Average CDS length (bp)	Average exon number per gene	Average exon length (bp)	Average intron length (bp)
denovo/AUGUSTUS	19,271	19,665.20	1,726.50	10.08	171.34	1,976.46
denovo/GlimmHMM	54,008	14,259.34	905.18	6.10	148.33	2,617.17
denovo/Genscan	23,400	24,954.02	1,692.64	9.19	184.09	2,838.60
homo/*C. carpio*	46,149	10,108.37	1,077.86	5.61	91.98	1,957.04
homo/*S. grahami*	43,803	11,026.80	1,115.46	5.75	193.90	2,085.45
homo/*M. amblycephala*	47,792	12,277.38	1,201.90	5.81	207.02	2,304.66
homo/*D. rerio*	45,504	9,494.07	1,020.30	5.28	193.18	1,979.17
homo/*C. idella*	63,196	7,385.67	972.24	4.59	211.79	1,786.17
trans.orf/RNAseq	15,467	21,165.74	1,680.38	10.78	281.86	1,853.98
PASA	24,038	19,597.60	1,651.11	9.97	257.30	1,898.72
MAKER	24,153	18,969.98	1,638.21	9.87	243.04	1,868.06

**Table 7:** Functional annotation statistics.

	Gene number	Percent (%)
Total	24,038	NA
InterPro	20,189	83.99
GO	14,812	61.62
KEGG_ALL	22,561	93.86
KEGG_KO	16,013	66.62
Swissprot	20,884	86.88
TrEMBL	22,382	93.11
NR	22,936	95.42
Annotated	22,965	95.54
Unannotated	1,073	4.46

### Annotation of non-coding RNA genes

The tRNAscan-SE (v1.3.1)^40^ algorithms with default parameters were used to identify the genes associated with tRNA. We downloaded the closely related species rRNA sequences from the Ensembl database. Then rRNAs in the database were aligned against our genome using BLASTn (v2.6.0)^41^ with E-value <1e-5, identity ≥85% and match length ≥50 bp. The miRNAs and snRNAs were identified by Infernal (v1.1.2)^42^ software against the Rfam (v14.1) database with default parameters. As a result, we annotated 76 rRNAs, 2469 tRNAs, 291 MiRNAs and 212 snRNAs (Table 8).

**Table 8:** Statistics of non-coding RNA annotation.

Type		Copy	AverageLength (bp)	TotalLength (bp)	% of genome
**miRNA**		291	88.84	25,853	0.0031
**tRNA**		2,469	75.51	186,428	0.0225
**rRNA**	rRNA	76	338.30	25,711	0.0031
	18 S	4	1,891.75	7,567	0.0009
	28 S	2	5,047.50	10,095	0.0012
	5 S	70	114.99	8,049	0.0010
**snRNA**	snRNA	212	128.75	27,295	0.0033
	CD-box	75	108.87	8,165	0.0010
	HACA-box	48	156.56	7,515	0.0009
	splicing	74	132.45	9,801	0.0012
	scaRNA	6	220.00	1,320	0.0002

## Data Records

All the raw sequencing data have been deposited in the NCBI database under the accession number SRP470306^43^. The genome assembly has been deposited at GenBank under the accession GCA_037101425.1^44^. Genome annotations, along with predicted coding sequences and protein sequences, can be accessed through the Figshare^45^.

## Technical Validation

The BUSCO was used to evaluate the quality of the genome assembly. We assessed assembly completeness using BUSCO (v3.0.259)^46^ with the reference arthropod gene set (n = 3,640). The final genome assembly showed a BUSCO completeness of 98.4%, consisting of 3,538 (97.2%) single-copy BUSCOs, 45 (1.2%) duplicated BUSCOs, 26 (0.7%) fragmented BUSCOs, and 31 (0.9%) missing BUSCOs (Table 9). Comparison of BUSCO results with *Squaliobarbus curriculus* (95.8%) and *Mylopharyngodon piceus* (96.0%) revealed the high genome assembly quality of yellow-cheeked carp^47^.

**Table 9:** Statistical result of BUSCO evaluation results of genome assembly.

	Number	Percentage (%)
Complete BUSCOs	3,583	98.4
Complete and single-copy BUSCOs	3,538	97.2
Complete and duplicated BUSCOs	45	1.2
Fragmented BUSCOs	26	0.7
Missing BUSCOs	31	0.9
Total BUSCO groups searched	3,640	100

## Data Availability

All commands and pipelines used in data processing were executed according to the manual and protocols of the corresponding bioinformatic software. No specific code has been developed for this study.
